# Evolution of ad hoc and routine data linkage in Australia

**DOI:** 10.23889/ijpds.v9i5.2815

**Published:** 2024-09-18

**Authors:** Erika Hagemann, Mikhalina Dombrovskaya, Felicity Flack

**Affiliations:** 1 The University of Western Australia; 2 Population Health Research Network

Australia has a rich history of utilising linked administrative data for health and human services research. The Population Health Research Network (PHRN), established in 2009, has developed national population-based data linkage capability and infrastructure. Australia-wide, specialist data linkage units have successfully linked their core population health data for over a decade, and within the network over 200 data collections are routinely linked. Data collections are typically classified as either routinely linked or linked on a project-by-project, ad hoc basis. Over time, linkage of data collections that were originally considered ad hoc, for example some longitudinal cohorts and clinical registries, has become ‘routine’.

This paper reports on findings of a study that evaluated the volume and proportion of data linkage requests that have been facilitated through the PHRN over the past 5 years that include ad hoc linkage. The paper also reports on the type of data collections that have been linked on an ad hoc basis and details the type of research projects and fields of research currently supported via ad hoc linkage. Quantifying and characterising the type and most sought-after data collections for ad hoc linkage and the breadth of research being undertaken informs future planning and resource allocation for expanding the Australian national linkage capability. This paper explores the integration of ad hoc data linkage within the evolving landscape of expanded master linkage files and national linked data assets, responding to a growing range of data sources and research that could benefit from population-based data linkage.

